# To Mask or Not to Mask—Evaluation of Cognitive Performance in Children Wearing Face Masks during School Lessons (MasKids)

**DOI:** 10.3390/children9010095

**Published:** 2022-01-11

**Authors:** Anne Schlegtendal, Lynn Eitner, Michael Falkenstein, Anna Hoffmann, Thomas Lücke, Kathrin Sinningen, Folke Brinkmann

**Affiliations:** 1 University Children′s Hospital, Ruhr-University Bochum, 44791 Bochum, Germany; lynn.eitner@rub.de (L.E.); anna.hoffmann-n28@rub.de (A.H.); thomas.luecke@rub.de (T.L.); kathrin.sinningen@rub.de (K.S.); folke.brinkmann@rub.de (F.B.); 2 ALA Institute, 44805 Bochum, Germany; falkenstein@ala-institut.de

**Keywords:** children, face masks, school, cognitive impairment, concentration

## Abstract

In the current SARS-CoV-2 pandemic, wearing a face mask is mandatory again during school lessons. There are no controlled studies in children to date indicating an effect on cognitive performance from wearing face masks. In a randomized controlled trial, we analysed the influence of face masks on cognitive performance of pupils during regular school lessons. Pupils (*n* = 133, fifth to seventh grade) were randomized by alternating allocation into control (with masks, *n* = 65) and intervention groups (without mask, *n* = 68). After two school lessons with (control) and without (intervention) face masks in class, all pupils performed digital tests for cognitive performance regarding attention and executive functions (switch, Corsi block-tapping, 2-back and flanker task). Overall, there were no significant differences in cognitive performance between both groups, masks vs. no masks. Wearing face masks has no significant influence on attention and executive functions of pupils and can still be recommended during school lessons.

## 1. Introduction

In the current SARS-CoV-2 pandemic, it is highly recommended to wear face masks to significantly reduce the spread of the virus [1]. In many areas, wearing a face mask is mandatory, including for school-age children during school lessons. So far, there are no studies that could prove a clinically relevant influence of masks in daily life, not even in young children [2]. Goh et al. examined end-tidal pCO_2,_ and oxygen saturation in 106 children aged 7–14 at rest and on exertion, finding no relevant hypoxemia or hypercapnia [3]. The same was demonstrated in three other studies showing that during physical exertion, all masks are safe and have only minimal impact on performance and physiological variables [4,5,6]. However, during high-intensity exercise, wearing a face mask can have a relevant influence [7]. Children wearing FFP2/K95 masks were more stressed, which was shown, for example, by a higher breathing rate or higher end-tidal CO_2_. However, other relevant parameters were not significantly affected (oxygen saturation or pulse rate), and the effect could probably be reduced by wearing a surgical mask [8]. 

There are no controlled studies in children to date indicating an effect on cognitive performance from wearing face masks. This could be a relevant problem especially in schoolchildren who have to wear the mask for hours during lessons. 

Therefore, we compared in a randomized controlled study the cognitive performance of schoolchildren wearing a face mask or no face mask for up to two hours during regular school lessons. 

## 2. Materials and Methods

### 2.1. Study Design 

We conducted a randomized, controlled intervention trial. In June 2021, participants were recruited in 5th, 6th, and 7th grade (13 school classes) at a comprehensive school in Gelsenkirchen, Germany, where prior trials with cognitive testing have successfully taken place with different classes [9,10,11,12]. The school has a focus on sports in some classes, for which children can apply for by passing a fitness test (Motor Test of North Rhine-Westphalia in Germany) [13]. Children with above-average scores in sporting performance are assigned to sport-focused classes (SC) with 5 h of physical education a week in the 5th grade and 6 h physical education in the 6th grade. Schoolchildren attending non-sport-focused classes (N-SC) have 3 h of physical education per week. All rooms used for the study had good ventilation facilities and were equipped with an air purifier. 

The study was approved by the Ethics Committee of the Medical Faculty of the Ruhr-University Bochum (Registration number 21-7218, dated 14 June 2021).

### 2.2. Participants and Randomization 

All pupils of the 5th and 6th grade (11 classes) and pupils of 2 classes of the 7th grade were asked to participate in the trial. Participants and their parents/guardians were informed by written information material and asked to sign the informed consent form if they wanted to take part. Exclusion criteria were missing signed consent form, positive SARS-CoV-2 testing, severe respiratory or neurological disease, and learning disorder. Furthermore, participants with incomplete cognition testing were excluded. Participants in each school class were randomised by alternating allocation to the intervention or control group. On the day of the study, all participants of the intervention group were tested for SARS-CoV-2 by rapid antigen test. At the end of the day, everyone received a small toy as a gift. 

### 2.3. Study Schedule

In the first two lessons, both groups (intervention and control groups) attended classes together wearing a face mask (FFP2/K95 or surgical mask). Before the third lesson, the two groups were separated in different rooms, and the intervention group took part in the next two lessons without a face mask. Teaching in the two groups was comparable. After these two lessons, cognitive assessment was done with (control group) and without wearing a face mask (intervention group). 

### 2.4. Cognitive Assessment

Cognitive performance was assessed in groups (intervention group = 5 to 11 children per group, control group 6 to 13 children per group) on individual computers using four computerized tasks that tested attention and executive functions developed by the ALA Institute in Bochum, Germany. Before the actual testing began, a pre-test was performed, which included the explanation of all tasks and a test trial for each task (duration approximately 1 min per task). Participants were asked to perform all tasks as quickly and accurately as possible. The tasks were applied in the following order:

#### 2.4.1. Switch Task

**Figure 1:**
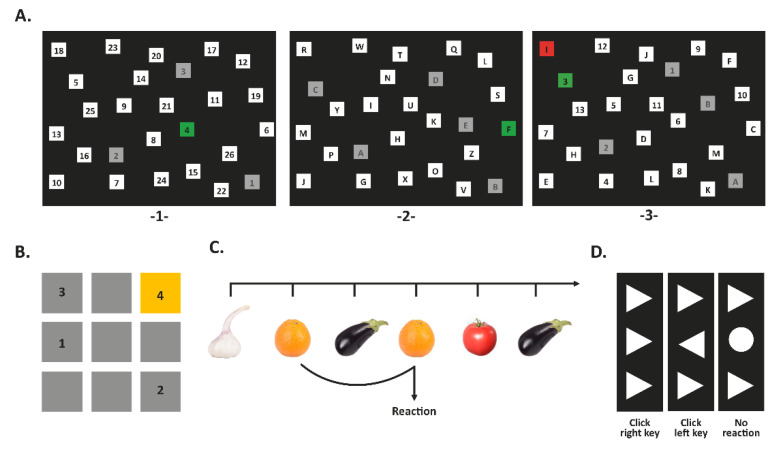
Computerized cognitive task. (**A**) Switch task: visual attention and task switching. The task comprised three sections. (-1-) First section, numbers (non-switch) had to be clicked in ascending order. (-2-) Second section, letters (non-switch) from A to Z had to be clicked alphabetically. (-3-) Third section, number and letters (switch) had to be clicked alternately in ascending order (i.e., 1,A,2,B,3,C). (**B**) Corsi block-tapping task: visual-spatial attention. A sequence of blocks lit up and gradually increased in length up to six blocks. Sequences had to be repeated. (**C**) 2-back task: working memory updating. Fruits and vegetables were displayed on a computer screen. A predefined key had to be pressed when the current image was the same as the image two trials back. (**D**) Flanker task: inhibitory control. Congruent flankers: click right key; incongruent flankers: click left key; no-go (circle): no reaction.

**Figure 2:**
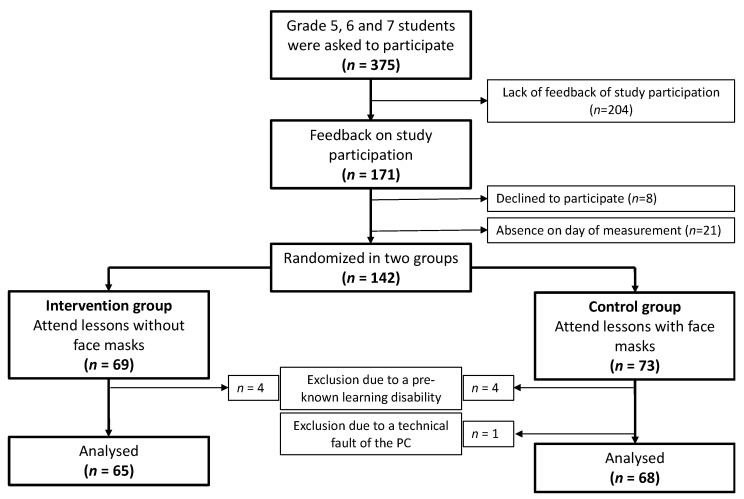
Flowchart—study design and population (*n* number).

Selective attention, search, and task switching were measured using an alternative version of the Trail Making Task consisting of three parts [9,10,11] (Figure 1). In the first section, black numbers from 1 to 26 in white squares were presented on a computer screen (Figure 1(A-1-)). Numbers had to be clicked in ascending order. If the numbers were clicked correctly, the squares turned green, otherwise red. Subsequently, correctly processed squares turned grey. The maximum time for processing was 3 min. The second section worked on the same principle, except that instead of numbers, letters from A to Z had to be put in the right order (Figure 1(A-2-)). In the third section, the 26 squares contained numbers from 1 to 13 and letters from A to M (Figure 1(A-3-)). Participants had to alternately click numbers and letters in ascending order (i.e. 1,A,2,B,3,C, etc.). Outcome parameters were reaction time (RT) for processing numbers (items 2–26), RT for processing letters (items 2–13), and switch costs (switch costs = switch RT [items 2–26]—numbers RT [items 2–26]—(letters RT [items 2–13]—numbers RT [items 2–13]). Negative switch costs indicating inadequate responses (at least one of the trials was not completed on time) were regarded as implausible and excluded from the analysis. 

#### 2.4.2. Corsi Block-Tapping Task (CORSI) 

CORSI assesses the capacity of the visuospatial subsystem within the working memory [10,11]. On the computer screen, a matrix of nine blue squares (3 by 3) was presented, which turned yellow in varying order and sequence length (three to six blocks; Figure 1B). Presented sequences had to be repeated by the participants. After every three sequences, the number of blocks increased by one. Each block highlighted in yellow for 500 ms with a 1000 ms interval between the blocks. After each sequence, the participants received feedback: a green arrow for correct answers, a red cross for incorrect answers. Main outcome parameters were the immediate block span (longest sequence correctly reproduced) and the number of correctly reproduced sequences. Additionally, the number of each correctly reproduced sequence was rated with a score. Depending on the sequence length, correctly reproduced sequences were weighed differently and multiplied with a factor ranging from 1 to 4: 3-block sequences × 1; 4-block sequence × 2; 5-block sequences × 3; 6-block sequence × 4. A maximum score of 30 could be achieved.

#### 2.4.3. 2-Back Task 

To assess working memory, the n-back task was used in a 2-back condition as described before [9,10,11]. Briefly, 106 consecutive pictures of fruits and vegetables were presented in the middle of the screen (Figure 1C). When the current picture matched the picture presented two trials earlier (*n* − 2), participants had to press a predefined computer key. The stimuli were presented for 500 ms (interstimulus interval: 2100 ms, maximal RT: 1400 ms). A green checkmark appeared on the screen for correct responses, and a red cross for incorrect responses. Twenty-one pictures were targets (same picture as 2 trials before). Main outcome parameters were mean RT, ratio of missing (no reaction while reaction was required), and ratio of false alarms (reaction while no reaction was required). 

#### 2.4.4. Flanker Task 

Flanker task measures the inhibitory control, i.e., the ability to suppress responses to a stimulus [10,11]. Three superposed triangles were presented on the computer screen. The upper and lower triangles were pointing in the same direction independently from the target in between. Each trial was categorized as compatible, incompatible, or no-go (Figure 1D). In compatible trials, flankers and the target were pointing in the same direction, in incompatible trials in opposite directions. In no-go trials, the target was replaced by a circle. The participants were instructed to press a left or a right key according to the target direction or not to react when the circle appeared. To induce flanker–target conflicts, flankers were presented individually for 100 ms and remained together with the target for another 100 ms. Maximal RT was 1100 ms, the response stimulus interval was 1000 ms (varying ±20%). If participants did not react within 600 ms, “faster” was displayed on the computer screen after the maximal RT had elapsed. In total, the task consisted of 102 items (32 no-go, 35 compatible, 35 incompatible). Outcome parameters were difference between mean RT of compatible and incompatible trials (RT slowing), difference between the ratio of compatible and incompatible trials (difference error rate), and count of false alarms. To avoid implausible data, negative RT slowing and negative difference error rates were excluded.

### 2.5. Statistical Analyses

All analyses were performed using the statistical software package IBM^®^ SPSS^®^ Statistics for Windows, version 25.0 (IBM Corp., Armonk, NY, USA). Interval-scaled parameters of the cognitive tasks were used as outcome variables (switch task: switch costs, visual search letters, visual search numbers; 2-back task: RT, ratio of missing, ratio of false alarms; CORSI: immediate block span, number of correct sequences, score; flanker task: RT slowing, difference error rate, count of false alarms). A power analysis with 80% power and α = 0.05 revealed that a sample size of 54 participants (dropout rate = 10%) was required to detect differences in cognitive performance between both groups. Descriptive data were analysed with the chi-squared test. Normally distributed data were analysed with Student’s *t*-test, non-normally distributed data were analysed with Mann–Whitney test. To overcome type I error due to multiple testing (number of outcome parameters *n* = 12), *p*-values were adjusted with Bonferroni correction. *p*-values < 0.05 were considered statistically significant, and effect size was calculated (*t*-test: $r=\surd \frac{t^{2}}{t^{2}+df}$; Mann–Whitney test: $r=Z/\sqrt{n\;}$). Data are presented as mean ± standard deviation (SD) or median (25th–75th interquartile range).

## 3. Results

### 3.1. Participants

A total of 142 students (their parents/guardians) agreed to participate in the study and completed the PC-based cognition test. Eight of them had to be excluded from analyses due to a pre-known learning disorder, and for one participant, cognition tests were incomplete, so that we were able to include a total of 133 students (see flowchart Figure 2). All SARS-CoV-2 tests of the students without face masks were negative, and no case of COVID-19 occurred in the classes during and after the study. All participants wore face masks during the first two lesson of the day. After the usual 15-minute break that followed, during which students are allowed to take of their face masks outside, students were randomized into one group with face mask (*n* = 73) and one without face mask (*n* = 69). The students wore the type of face mask they would normally wear at school. Since only 6 of the 73 students in the group with face mask wore a K95/FFP2 mask during the study, we did not analyse this group separately. Participant’s characteristics are shown in Table 1.

### 3.2. Cognition

There were a few children who did not complete one of these tests (one participant of the +Mask group was missing for Corsi block-tapping task, nine children (−Mask: 6; +Mask: 3) for task-switching analysis because of negative switch costs, and 24 for flanker task (−Mask: 8; +Mask: 16) because of negative RT slowing and difference error rate). The range (min–max) of the cognition parameters tested is shown in Table S1 in the supplement section and was comparable between the −Mask and +Mask groups.

### 3.3. Wearing Masks and Cognition

Statistical analysis revealed no differences in cognitive performance between both groups, masks vs. no masks, regarding switch costs, visual search letters, and visual search numbers of the switch task (Table 2). Similarly, the processing time for the 2-back tasks was not significantly different, and the error rate was comparable between the two groups as well. The ratio of missings tended to be higher in the mask-wearing group with 3.81% (2.62–5.24) compared to 3.10% (2.02–4.29) in the group not wearing any masks (r = 0.19; *p* = 0.03). However, these differences were not statistically significant after Bonferroni correction (*p* = 0.36). In contrast, cognitive performance in terms of visuospatial memory analysed with the Corsi block-tapping task was almost identical. Both groups were able to correctly repeat a sequence length of five blocks on average with a total of seven correct sequences. Accordingly, a comparable score was achieved in both groups. Inhibitory control revealed no differences with regard to RT slowing and the difference error rate between participants wearing a mask and those not wearing a mask. Count of false alarms tended to be higher in the +Mask group (7.00 (3.50–18.0)) compared to the –Mask group (3.00 (1.00–8.75)) (r = 0.15; *p* = 0.006). However, these differences were significant only when type I error was neglected (*p* = 0.07).

### 3.4. Wearing Masks and Cognition: Non-Sport-Focused Classes (N-SC) vs. Sport-Focused Classes (SC)

With regard to the switch task, children attending SC were faster compared to children from N-SC; however, we found no differences in RT of switch costs, visual search letters, and visual search numbers between the −Mask group and +Mask group, neither in N-SC nor SC (Table S3). The processing time of the 2-back task was comparable between both groups in children from N-SC, while children from SC without masks took 490 ± 125 s compared to 538 ± 81.1 s with masks. However, these differences were not statistically significant. Visuospatial memory was not affected by mask wearing, in neither N-SC nor SC. Inhibitory control of N-SC and SC revealed no differences in RT slowing and difference error rate between participants wearing a mask and those not wearing a mask. In children attending N-SC, count of false alarms tended to be higher in the +Mask group (6.50 (3.00–15.5)) compared to the −Mask-group (2.50 (1.00–5.25)) (r = 0.2; *p* = 0.005). However, these differences were not statistically significant after Bonferroni correction (*p* = 0.06).

### 3.5. Wearing Masks and Cognition: Age-Specific Differences

When looking at the cognitive performance of pupils in the fifth, sixth, and seventh grade individually, no differences could be detected (Table 3). Concerning the 2-back task, participants attending the 5th grade tended to have an increased RT when wearing a face mask, while seventh graders had gradually more missings (37.0% ± 18.8) compared to those not wearing a mask (25.8% ± 12.5). On the contrary, count of false alarms in the flanker task tended to be increased after wearing a mask (r = 0.18, *p* = 0.04). However, again, this was only the case when type I error was neglected (*p* = 0.48).

## 4. Discussion

Face masks are effective in reducing transmission of SARS-CoV-2 and other aerogenic pathogens [14]. Especially indoors, they offer better protection against transmission compared to not wearing masks [15]. Since the reopening of schools during the pandemic, pupils in Germany areobliged to wear face masks (surgical or FFP2) in class, and children and adolescents, as well as adults, have become well accustomed to wearing face masks in class and at work. In adults, adverse effects of wearing face masks such as hypoxemia, hypercapnia, dyspnoea, and neurological symptoms including headache, drowsiness, and dizziness have been described [16]. In a large survey of approximately 25,000 children wearing a face mask for an average of 270 min, 68% were impaired, reported by their parents. Complaints included irritability (60%), headache (53%), difficulty concentrating (50%), less happiness (49%), reluctance to go to school/kindergarten (44%), malaise (42%), impaired learning (38%), and drowsiness or fatigue (37%) [17]. But these data were obtained by interviewing parents and doctors alone, without a control group. There is no indication that wearing a face mask impairs any physiological variables such as oxygen saturation or end-tidal CO_2_ in children and adolescents, even during physical activity [2,3], although this was initially stated by a now retracted paper by Walach et al. [18].

Concerns remain that wearing face masks reduces the ability to concentrate on tasks during school lessons. This is the first study evaluating the effect of wearing a face mask on cognition and the ability to concentrate in a real-life setting. 

We used a computer-based test tool that has proven in previous studies to be very sensitive regarding mild cognitive impairment, e.g., by changes in water supply [10].

The effects on concentration and cognition were evaluated in children aged approximately 11–14 years from fifth to seventh grade who were randomized to wearing or not wearing a face mask in class. Comparing the two groups, the ranges of the measured parameters were comparable in both groups, and we could not find any significant differences in the various cognition tests. Wearing a face mask had no effect on the students′ cognitive performance in our study. Relevant influences of age or physical activity of the students were not detectable. Admittedly, however, we observed tendencies regarding a slightly worse cognitive performance in the group of mask wearers. This tendency to differ in performance can possibly be explained by the unequal distribution of the groups. The proportion of fifth graders was slightly higher in the +Mask group compared to the −Mask group. Although there is only an approximately one-year age difference between fifth and sixth graders, their cognitive performance differs significantly, as we have shown before [11]. On the other hand, cognition seems to be age-dependent in all cognitive domains and not only in a single parameter such as the ratio of missings in the 2-back task as it appeared in this study. Next to age, physical fitness can also be a contributor to cognitive performance. An advanced level of physical education at school, and thus an increased physical activity, seems to be associated with overall better cognitive performance, specifically in response speed and accuracy [11]. In this study, the proportion of children from N-SC and SC was approximately equal in the control and intervention groups so that physical fitness should have not affected the outcome. However, when analysing N-SC and SC separately, it showed that especially pupils from SC were not affected at all from wearing a mask. Only fifth graders tended to show worse cognition after wearing a mask as long as type I error was neglected. Overall, these data suggest that wearing a mask has no effect on schoolchildren′s cognition. Possible tendencies can certainly be neglected, especially because effect sizes were very small.

### Limitation

An important limitation of the study is certainly the short time span of only two school hours plus the time of testing (about one school hour), during which the students of the intervention group did not wear a mask. The students with a face mask, however, had been wearing them for four lessons at the time of testing. During the first two lesson in the morning, all students had to wear a face mask. Therefore, no statement can be made about the influence on concentration performance by mask wearing over longer periods of time. For organisational reasons (e.g., necessary teaching staff for both groups and additional rooms for separate lessons for the two groups), a longer period during ongoing school operations was not possible. In Germany, however, the duration of two school hours corresponds exactly to the lesson unit, after which pupils have a longer break (15–20 min) to regenerate outside without a face mask, so that the chosen duration reflects the real-life situation of one day at school quite well.

Fitting of the face masks in the control group was not systematically checked and corrected. The students wore the mask as they do in everyday school life. However, again, this is in line with the real-life setting. 

As the study took place during ongoing school operations, no block randomisation was possible for organisational reasons. Nevertheless, we were able to achieve a relatively even distribution of the students among the groups.

Similarly, no distinction was made between surgical masks and FFP2/KN95 masks in the evaluation, as otherwise the sample sizes, especially in the group of students wearing K95/FFP2 masks, would have been too small. There are indications in studies that tighter-fitting masks stress subjects more, but without seriously affecting them [3,8]. In addition, in the “Co-Ki” survey [17], the majority of children reported wearing looser-fitting masks such as cloth masks or surgical masks, so we do not suspect a major influence. 

Possible side effects of the masks such as discomfort, headaches, and breathing problems were not systematically recorded. However, none of the participants complained of discomfort during the study, and none had to stop the testing early. 

Drinking and eating were not regulated during the study. We assume, however, that we were able to sufficiently reduce the influence of this aspect through randomisation within the school classes. 

Another limitation is the lack of data on possible communication and language deficits during school lesson that could be caused by wearing face masks as described by other authors [19]. Our study was only a short-term intervention with focus on cognitive function. These aspects would have to be considered in further studies.

## 5. Conclusions

We were able to show in our study that wearing a face mask has no influence on the cognitive performance of pupils. 

From our point of view, wearing of face masks in class during the pandemic can still be recommended and should become as self-evident as wearing a helmet when cycling or buckling up in a car while driving.

## Figures and Tables

**Table 1:** Demographic data: size of groups by grade level, sex and sport-focused class.

	−Mask	+Mask	*p*
Total, *n* (%)	68	65	
Girls, *n* (%)	42 (61.8)	30 (46.2)	0.07
Grade 5, *n* (%)	23 (33.8)	37 (56.9)	0.07
Grade 6, *n* (%)	28 (41.2)	15 (23.1)	0.05
Grade 7, *n* (%)	17 (25.0)	13 (20.0)	0.53
Children from sport-focused class *n* (%)	33 (48.5)	34 (52.3)	0.66

*n,* number; *p,* group-specific level of significance; significant at *p* < 0.05.

**Table 2:** Cognitive performance of children without (−Mask) and with a mask (+Mask).

	−Mask	+Mask	*p*	p *
Switch Task	*n* = 62	*n* = 62		
Switch costs (s)	27.2 ± 18.1	28.6 ± 18.2	0.68	1.00
Visual search letters (s) ^#^	34.8 (30.0–42.3)	36.7 (31.6–43.5)	0.25	1.00
Visual search numbers (s)	50.1 (43.6–58.9)	49.5 (43.7–58.4)	0.68	1.00
2-Back Task	*n* = 68	*n* = 65		
RT (ms)	512 ± 108	534 ± 88.7	0.18	1.00
Ratio of missings (%)	31.0 (20.2–42.9)	38.1 (26.2–52.4)	0.03	0.36
Ratio of false alarms (%)	8.24 (4.71–12.9)	9.41 (5.88–21.2)	0.29	1.00
Corsi Block-Tapping Task	*n* = 68	*n* = 64		
Immediate block span (*n*)	5.00 (5.00–6.00)	5.00 (5.00–6.00)	0.87	1.00
Correct sequences (*n*)	7.00 (5.00–8.00)	7.00 (5.00–8.00)	0.73	1.00
Score	12.0 (9.00–17.0)	12.5 (9.00–17.8)	0.84	1.00
Flanker Task	*n* = 60	*n* = 49		
RT slowing (ms)	75.2 ± 33.9	74.6 ± 43.8	0.93	1.00
Difference error rate (%)	19.4 (10.1–50.1)	35.5 (14.2–73.4)	0.12	1.00
Count of false alarms (*n*)	3.00 (1.00–8.75)	7.00 (3.50–18.0)	0.006	0.07

Normally distributed data are presented as mean ± standard deviation, non-normally distributed are displayed as median (25th–75th percentile); ^#^ first twelve reactions; *n,* number; ms, milliseconds; RT, reaction time; s, second; * Bonferroni-corrected. Ranges (min–max) of cognitive performance of children without (−Mask) and with a mask (+Mask) as well as reference values of the tests are shown in the supplement.

**Table 3:** Cognitive performance of children without (−Mask) and with a mask (+Mask)—allocation by grade level.

	5th Grade				6th Grade				7th Grade			
	−Mask	+Mask	*p*	*p* *	−Mask	+Mask	*p*	*p* *	−Mask	+Mask	*p*	*p* *
Switch Task	*n* = 20	*n* = 35			*n* = 26	*n* = 14			*n* = 16	*n* = 13		
Switch costs (s)	30.9 (19.8–46.4)	23.6 (17.0–40.4)	0.38	>0.99	22.9 ± 12.5	27.3 ± 15.7	0.33	>0.99	24.3 ± 16.5	25.0 ± 19.2	0.92	>0.99
Visual search letters (s) ^#^	36.0 (30.5–43.4)	38.5 (31.6–46.3)	0.50	>0.99	36.5 (31.4–44.0)	34.2 (31.4–43.0)	0.75	>0.99	31.1 (27.5–38.8)	36.5 (30.1–41.1)	0.22	>0.99
Visual search numbers (s)	54.1 ± 15.7	54.7 ± 14.7	0.90	>0.99	53.6 ± 12.2	48.1 ± 8.92	0.15	>0.99	48.7 7.75	48.7 ± 13.1	> 0.99	>0.99
2-back task	*n* = 23	*n* = 37			*n* = 28	*n* = 15			*n* = 17	*n* = 13		
RT (ms)	510 ± 84.2	554 ± 88.6	0.06	0.72	500 ± 135	511 ± 85.3	0.76	>0.99	550 (457–593)	540 (430–562)	0.34	>0.99
Ratio of missings (%)	36.2 ± 20.9	43.5 ± 20.8	0.19	>0.99	33.3 (23.8–42.9)	38.1 (14.3–47.6)	0.65	>0.99	25.8 ± 12.5	37.0 ± 18.8	0.06	0.72
Ratio of false alarms (%)	10.6 (4.71–22.6)	9.41 (5.88–21.2)	0.93	>0.99	7.65 (5.88–11.5)	9.41 (1.18–20.0)	0.90	>0.99	9.41 (4.71–12.4)	10.6 (5.29–27.7)	0.30	>0.99
Corsi block tapping task	*n* = 23	*n* = 37			*n* = 28	*n* = 14			*n* = 17	*n* = 13		
Correct immediate block span (*n*)	5.00 (4.00–6.00)	5.00 (4.00–6.00)	0.45	>0.99	5.00 (5.00–6.00)	5.50 (5.00–6.00)	0.74	>0.99	6.00 (5.00–6.00)	5.00 (5.00–6.00)	0.87	>0.99
Correct sequences (*n*)	5.70 ± 2.08	6.05 ± 2.09	0.52	>0.99	7.00 (6.00–8.00)	7.50 (6.00–9.00)	0.89	>0.99	8.00 (5.50–9.00)	8.00 (6.50–9.00)	0.77	>0.99
Score	9.00 (6.00–13.0)	11.0 (7.00–16.0)	0.37	>0.99	14.6 ± 5.54	14.4 ± 5.12	0.52	>0.99	15.5 ± 5.94	16.6 ± 5.70	0.60	>0.99
Flanker task	*n* = 20	*n* = 27			*n* = 24	*n* = 12			*n* = 16	*n* = 10		
RT slowing (ms)	58.2 ± 28.1	71.3 ± 48.2	0.25	>0.99	91.4 ± 38.4	71.4 ± 35.2	0.14	>0.99	72.2 21.1	87.1 42.2	0.32	>0.99
Difference error rate (%)	34.6 (14.2–71.6)	35.5 (15.6–89.2)	0.61	>0.99	22.9 (10.3–58.4)	45.8 (13.9–75.6)	0.28	>0.99	13.3 (6.64–36.7)	21.0 (5.74–81.6)	0.59	>0.99
Count of false alarms (*n*)	5.50 (2.25–18.0)	11.0 (3.00–19.0)	0.28	>0.99	3.50 (1.00–11.3)	7.50 (4.25–16.8)	0.04	0.48	2.00 (1.00–5.75)	4.50 (1.75–7.25)	0.22	>0.99

Normally distributed data are presented as mean ± standard deviation, non-normally distributed are displayed as median (25th–75th percentile); ^#^ first twelve reactions, n, number; ms, milliseconds; RT, reaction time; s, second; * Bonferroni-corrected; significant *p* < 0.05.

## Data Availability

The data presented in this study are available on request from the corresponding author.

## Supplementary Materials

The following are available online at https://www.mdpi.com/article/10.3390/children9010095/s1, Table S1: Ranges (min–max) of cognitive performance without (−Mask) and with a mask (+Mask). Table S2: Reference values of cognitive performance—mean value (MV) and median including 25th–75th percentile. Table S3: Cognitive performance of children from non-sport-focused classes (N-SC) and sport-focused classes (SC) without (−Mask) and with a mask (+Mask).
