# Supplementary material for: To Mask or Not to Mask—Evaluation of Cognitive Performance in Children Wearing Face Masks during School Lessons (MasKids)
Source: Children (Basel). 2022 Jan 11;9(1):95. doi: 10.3390/children9010095 (PMC8774884; doi:10.3390/children9010095)
Supplement: Supplementary file 1 [file children-09-00095-s001.zip › children-1513571-supplementary.pdf]

Table S1. Ranges (min-max) of cognitive performance without (– Mask) and with a mask (+ Mask).

	**– Mask**	**+ Mask**
Switch Task	n = 62	n = 62
Switch costs (s)	1.00-92.7	1.78-78.2
Visual search letters (s)[#]	19.9-67.9	19.7-108
Visual search numbers (s)	35.2-98.1	30.6-106
2-back task	n = 68	n = 65
RT (ms)	111-720	357-789
Ratio of missings (%)	4.76-85.7	4.76-95.2
Ratio of false alarms (%)	0-73	0-67
Corsi block tapping task	n = 68	n = 64
Immediate block span (n)	4-6	4-6
Correct sequences (n)	3-12	1-12
Score	4-30	2-30
Flanker task	n = 60	n = 49
RT slowing (ms)	4-195	0-208
Difference error rate (%)	0-308	0-391
Count of false alarms (n)	0-24	0-32

[#] first twelve reactions, n number, ms milliseconds, RT reaction time, s second

Table S2. Reference Values of cognitive performance

	MV ± SD	Median (25th-75th percentile)
Switch Task[1]	n = 849	
Switch costs (s)	30.9 ± 19.9	27.4 (16.5-41.4)
Visual search letters (s)[#]	37.7 ± 13.2	34.6 (28.9-42.5)
Visual search numbers (s)	54.0 ± 13.8	51.4 (44.4-60.7)
2-back task[1]	n = 946	
RT (ms)	481 ± 124	476 (391-561)
Ratio of missings (%)	33.3 ± 18.1	33.3 (19.1-42.9)
Ratio of false alarms (%)	18.8 ± 21.0	9.41 (4.71-23.5)
Corsi block tapping task[2]	n = 563	
Immediate block span (n)	5.17 ± 0.83	5.00 (5.00-6.00)
Correct sequences (n)	6.75 ± 2.48	7.00 (5.00-8.00)
Score	12.3 ± 6.14	12.0 (9.00-17.0)
Flanker task[2]	n = 417	
RT slowing (ms)	76.3 ± 38.8	73.4 (53.2-97.3)
Difference error rate (%)	37.7 ± 56.4	22.1 (10.0-41.6)
Count of false alarms (n)	9.19 ± 7.26	7.00 (3.00-15.0)

Mean Value (MV) and Median including 25th-75th Percentile. [#] first twelve reactions, n number, ms milliseconds, RT reaction time, s second, SD standard deviation. [1] Merged cognition data from the following studies: Jansen et al. 2020 [9], Drozdowska et al. 2020 [10,11], Drozdowska et al. 2021 [19], Sinningen et al. (ongoing study of the University children´s hospital, Ruhr-University Bochum, unpublished data); [2] Merged cognition data from the following studies: Drozdowska et al. 2020 [10,11], Drozdowska et al. 2021 [19]. Only 5th and 6th grade students were included.

Table S3. Cognitive performance of children from non-sport focused classes (N-SC) and sport-focused classes (SC) without (– Mask) and with a mask (+ Mask).

	**N-SC**				**SC**			
	– Mask	+ Mask	p	p*	– Mask	+ Mask	p	p*
Switch Task	n = 33	n = 30			n = 29	n = 32		
Switch costs (s)	27.2 ± 18.9	30.2 ± 19.9	0.54	> 0.99	24.1 (18.0-30.6)	22.2 (15.4-40.2)	0.91	> 0.99
Visual search letters (s)	37.2 (30.6-42.7)	37.6 (31.3-44.0)	0.39	> 0.99	33.0 (29.7-41.6)	36.2 (31.7-44.0)	0.24	> 0.99
Visual search numbers (s)	53.8 ± 11.7	51.3 ± 10.9	0.52	> 0.99	49.6 (40.2-56.2)	46.7 (43.1-57.7)	0.91	> 0.99
2-back task	n = 35	n = 31			n = 33	n = 34		
RT (ms)	532 ± 86.2	531 ± 97.6	0.97	> 0.99	490 ± 125	538 ± 81.1	0.07	0.84
Ratio of missings (%)	32.9 ± 18.9	40.1 ± 21.3	0.15	> 0.99	28.6 (23.8-42.9)	38.1 (27.4-47.6)	0.09	> 0.99
Ratio of false alarms (%)	8.24 (4.71-10.6)	10.6 (4.71-24.7)	0.08	0.96	9.41 (5.88-20.0)	9.41 (5.88-19.1)	0.81	> 0.99
Corsi block tapping task	n = 35	n = 31			n = 33	n = 33		
Correct immediate block span (n)	5.00 (5.00-6.00)	5.00 (5.00-6.00)	0.72	> 0.99	5.00 (4.00-6.00)	5.00 (5.00-6.00)	0.58	> 0.99
Correct sequences (n)	7.00 (5.00-8.00)	6.00 (5.00-8.00)	0.93	> 0.99	7.00 (6.00-8.00)	7.00 (5.00-8.00)	0.75	> 0.99
Score	12.0 (9.00-18.0)	12.0 (9.00-18.0)	1.00	> 0.99	13.5 ± 6.03	12.9 ± 5.80	0.52	> 0.99
Flanker task	n = 30	n = 22			n = 30	n = 27		
RT slowing (ms)	77.4 ± 24.2	71.7 ± 50.5	0.63	> 0.99	73.1 ± 41.7	76.8 ± 38.2	0.73	> 0.99
Difference error rate (%)	16.2 (7.06-43.6)	29.8 (7.72-58.7)	0.15	> 0.99	30.9 (13.7-76.6)	39.9 (14.6-80.2)	0.59	> 0.99
Count of false alarms (n)	2.50 (1.00-5.25)	6.50 (3.00-15.5)	0.005	0.06	5.00 (1.75-21.3)	8.00 (4.00-19.0)	0.34	> 0.99

Normally distributed data are presented as mean ± standard deviation, non-normally distributed are displayed as median (25th-75th percentile), n number, ms milliseconds, RT reaction time, s second, *Bonferroni-corrected, significant p < 0,05
